# Supplementary material for: Development of an RNA aptamer-assisted CRISPR/Cas9 system for efficiently generating and isolating Cas9-free mutants in plant
Source: PLoS Genet. 2025 Nov 13;21(11):e1011931. doi: 10.1371/journal.pgen.1011931 (PMC12614593; doi:10.1371/journal.pgen.1011931)
Supplement: S1 Fig — (a) MFE secondary structure of 3WJ-4 × Bro. (b) MFE secondary structure of 3WJ-8 × Bro.(c) MFE secondary structure of 3WJ-12 × Bro. (DOCX) [file pgen.1011931.s001.docx]

**Fig. S1**

**
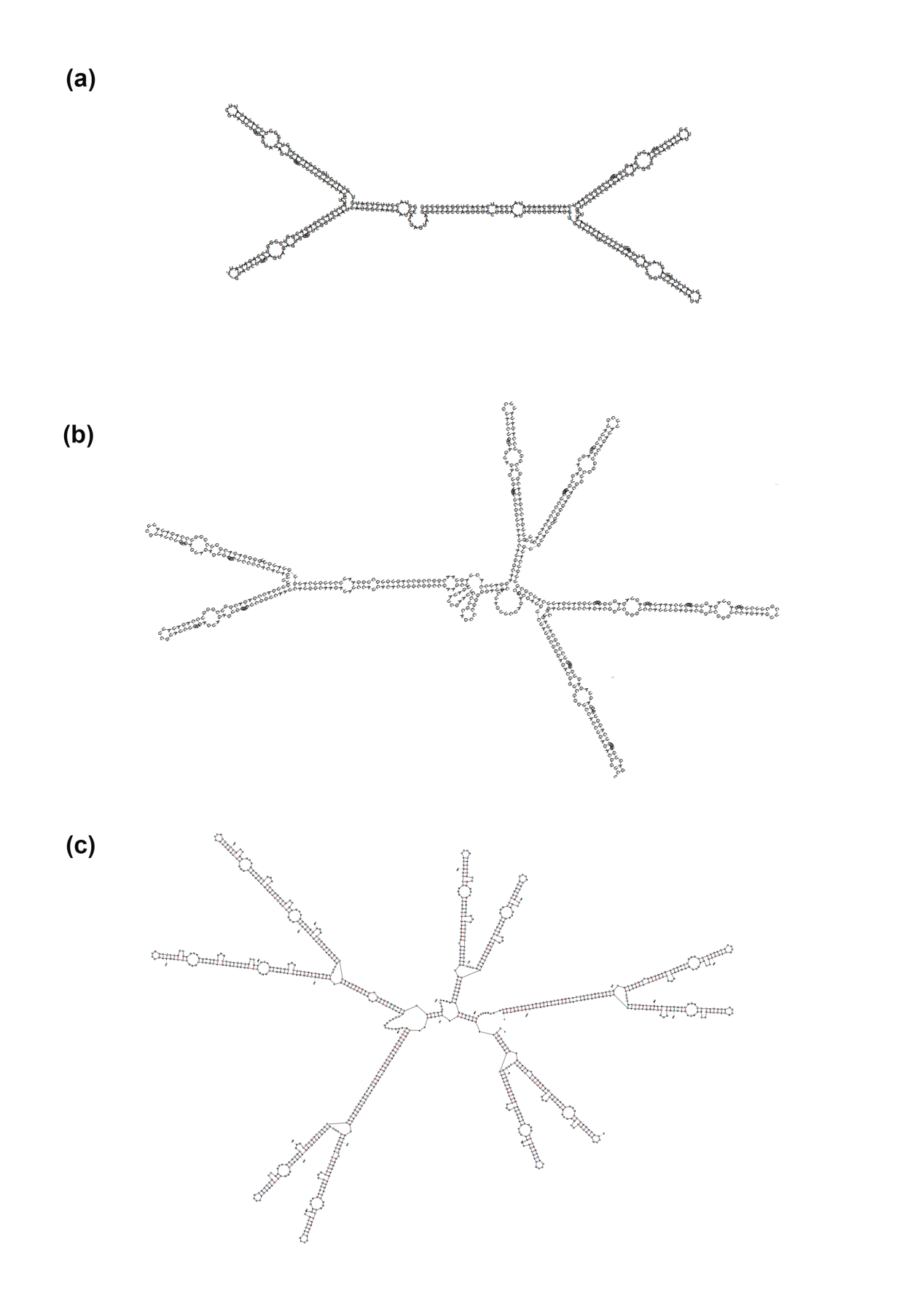
**

**Fig. S1** **Minimum free energy (MFE) secondary structure of 3WJ-n×Broccoli fluorescent aptamer.** **(a)** MFE secondary structure of 3WJ-4×Bro.**(b)** MFE secondary structure of 3WJ-8×Bro.**(c)** MFE secondary structure of 3WJ-12×Bro.
