# Supplementary material for: Development of an RNA aptamer-assisted CRISPR/Cas9 system for efficiently generating and isolating Cas9-free mutants in plant
Source: PLoS Genet. 2025 Nov 13;21(11):e1011931. doi: 10.1371/journal.pgen.1011931 (PMC12614593; doi:10.1371/journal.pgen.1011931)
Supplement: S2 Fig — (a) Rosette leaf area. (b) Number of rosette leaves. Error bars represent mean ± SD (n = 10). Statistical significance among multiple groups was determined using one-way ANOVA followed by Tukey’s post hoc test. ns, not significant. (DOCX) [file pgen.1011931.s002.docx]

**Fig. S2**


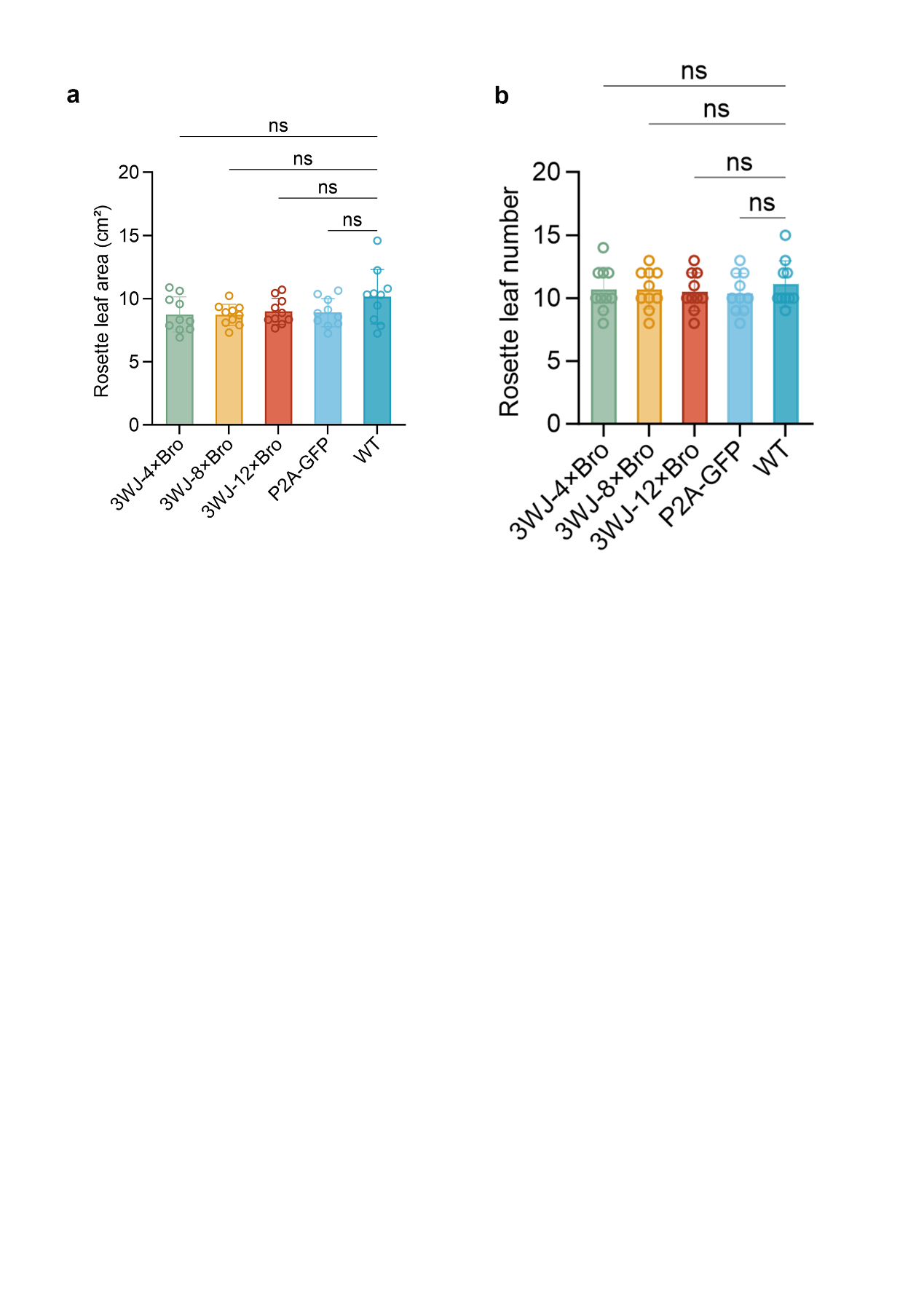


**Fig. S2 Growth status of transgenic *Arabidopsis* seedlings. (a)** Rosette leaf area. **(b)** Number of rosette leaves. Error bars represent mean ± SD (n = 10). Statistical significance among multiple groups was determined using one-way ANOVA followed by Tukey’s post hoc test. ns, not significant.
