# Supplementary material for: Development of an RNA aptamer-assisted CRISPR/Cas9 system for efficiently generating and isolating Cas9-free mutants in plant
Source: PLoS Genet. 2025 Nov 13;21(11):e1011931. doi: 10.1371/journal.pgen.1011931 (PMC12614593; doi:10.1371/journal.pgen.1011931)
Supplement: S1 Table — (DOCX) [file pgen.1011931.s003.docx]

**Supplementary Table 1**

Primers used for gene expression analysis and vector construction in this study.

| **Primer** | **Sequence (5' to 3')** |
| --- | --- |
| M13-F | gtaaaacgacggccagt |
| M13-R | gtcatagctgtttcctg |
| 4B-F | gcaaaaaagaaaaagaagcttTGATTGTCATGTGTATGTTGGGGAGA |
| 4B-R | ctctgagctcttacttgtacaCGGCCGCCAGTGTGATGG |
| 8B-F | gcaaaaaagaaaaagaagcttTGATTGTCATGTGTATGTTGGGG |
| 8B-R | ctctgagctcttacttgtacaCGCGATGAATCATCCAGCC |
| 12B-F | gcaaaaaagaaaaagaagcttTGATTGTCATGTGTATGTTGGGG |
| 12B-F | ctctgagctcttacttgtacaGAGCTCCGCGATGAATCATC |
| CX-F | CACTACCTGGACGAGATCATC |
| CX-R | CTCATATTAACTTCGGTCATTAGAG |
| U6-TT4-F | GATTGCCTAAGCTAGGCAAAGAAG |
| U6-TT4-R | AAACCTTCTTTGCCTAGCTTAGGC |
| U6-AtTTG1-T1-F | GATTGCCATATCCACTCTACGCCA |
| U6-AtTTG1-T1-R | AAACTGGCGTAGAGTGGATATGGC |
| U6-AtTTG1-T2-F | GATTGCCGGTCACAGAATCGCCGT |
| U6-AtTTG1-T2-R | AAACACGGCGATTCTGTGACCGGC |
| Bro-cx-F | AGCACCAAAGAGGTGCTGGA |
| Bro-cx-R | CACAGTTCGATAGCGAAAACCG |
| TT4-edi-F | ACATGACCGACCTCAAGGAG |
| TT4-edi-R | CATGAGACGCTTGACGGAAG |
| TTG1-edi-F | AAATCCGACTGACACTGACC |
| TTG1-edi-R | AAAACCCTAGCTTCTCCCCA |
| actin-F | CTTGAAACAGCAAAGACCAGC |
| actin-R | CATCCTATCAGCAATGCCCG |
| Cas9-F | AACCTATGCCCACCTGTTCG |
| Cas9-R | AGGATTGTCTTGCCGGACTG |
