# Supplementary material for: Development of an RNA aptamer-assisted CRISPR/Cas9 system for efficiently generating and isolating Cas9-free mutants in plant
Source: PLoS Genet. 2025 Nov 13;21(11):e1011931. doi: 10.1371/journal.pgen.1011931 (PMC12614593; doi:10.1371/journal.pgen.1011931)
Supplement: Attachment — Submitted filename: Cas9 Reviewer Comments_08102025.docx [file pgen.1011931.s005.docx]

In this manuscript, Liu et al. detail the development and evaluation of a new CRISPR/Cas9 system for plant gene editing. The authors introduce the 3WJ-4×Bro/Cas9 system, which utilizes an RNA aptamer as a transcriptional reporter to address challenges in screening for Cas9-free edited plants. The study directly compares this new system to the conventional GFP/Cas9 method, demonstrating its superiority in transformation efficiency, mutation rate, and the accurate identification of transgene-free mutants. The manuscript follows up on previous work done by the lab in developing the 3WJ-4bro aptamer. While the data is promising and has potential for application in plant biology and beyond, addressing the below enumerated concerns would bolster the narrative of this manuscript.

Figure 1

The diagram depicting aptamer fusion (1a) is not self-explanatory and the legend does not describe the diagram. The narrative describing Fig 1e and the figure legend for it are different. Fig 1e to me does not look like a graph that shows thermal stability or degradation of aptamers at different temperatures, rather a snapshot at one condition. It would help if the authors could clarify what the figure shows and how it relates to thermal stability of the aptamers. In addition, it would be helpful to have the individual datapoints represented in all bar graphs.

Figure 2

The authors have tried to characterize performance of the RAA/Cas9 system in *N Bethamiana* but this is a transient transformation and would be mosaic. Extracting total RNA and total protein from mosaic expressing plants could confound the amount of cas9 and may account for the differences in cas9 shown by immunoblotting. Perhaps this requires some clarification in the methods and results section of this manuscript.

The color scheme used for the bar chart here is different to Fig 1. Maintaining a uniform color scheme for each condition across all figures will help the reader.

Figure legend says they used an n=3 for expression studies. Are these 3 different plants? Or 3 leaves from the same plant? Did each experiment have technical replicates?

Figure 3

The experimental design for screening transformants is rigorous and the use of multiple assays allows for the identification of false positive and false negative screens. Statistical analysis of this screen is depicted in Fig 3b however, this figure graph is not described comprehensively in the figure legend and statistical significance is not denoted. I would like to request that the authors maintain consistency in their labeling and color scheme which would help bring their point across easily. Figures 3c.d, e, and f should have the order of conditions and colors per condition consistent among them. The authors report that growth of Arabidopsis plants is not affected by the RAA or GFP based cas9 systems, but they have not reported the parameters measured, any quantifications or even the number of plants they used to draw this conclusion. Perhaps this could be clarified in the figure legend and by including it in the methods.

Figure 4

The chromatogram for homozygous mutation shows large deletion with most of the base pairs proximal to the PAM sequence. Two of the other genotyped examples shown here illustrates insertions or deletions before the PAM sequence. While this is possible, it is thought to be less frequent. Did the authors see this phenomenon in most of their mutants? if so what does it mean for the application of this technology in plant genome engineering?

Figure 5

Authors have attempted to investigate the efficiency of isolating cas9-free mutants in the T2 generation. However, the rationale for using dual gRNAs is not clear and the conclusions drawn from this figure does not shed light on whether dual gRNAs are necessary for RAA/Cas9 system. When you say editing of Att4 results in trichome defects at T1- is this 100% and shown in all T1 plants.

Furthermore, they use Fig 5c to make two different points. 1) While talking about T1 they mention - Fig 5c 3WJ-4Bro/Cas9 induces homozygous mutations at both target sites, but GFP/Cas9 only in 1. 2) They point to Fig 5c to illustrate their point that only 1 homozygous mutant for both sites was found in cas9 Free T2 population. I am confused about which point 5c illustrates. If only one Homozygous mutant for both sites was found – then point 1 is not well supported. Authors should represent point 1 with other chromatogram examples. Fig 5c can only represent T1 or T2 – not both.

Discussion- Is only a reiteration of the results and lacks in-depth discussion about the biochemistry of these aptamers and applications in the field. This section requires some work.

Statistical Analysis- You have opted to use a Student’s T test even when you are comparing more than two independent variables. Why did you opt for this instead of a One Way ANOVA. Please expand on how you did the statistical analysis in the methods section. Some legends don’t have sample sizes.

Figures - Please make the x axis label order of samples consistent across all figures and maintain a consistent color scheme for the samples. Show us the individual datapoints for the bar graphs.
