# Supplementary material for: Development of an RNA aptamer-assisted CRISPR/Cas9 system for efficiently generating and isolating Cas9-free mutants in plant
Source: PLoS Genet. 2025 Nov 13;21(11):e1011931. doi: 10.1371/journal.pgen.1011931 (PMC12614593; doi:10.1371/journal.pgen.1011931)
Supplement: Attachment — Submitted filename: Response to Reviewers.docx [file pgen.1011931.s007.docx]

Dear reviewers,

Thank you for your careful review and constructive suggestions regarding our manuscript entitled *“Development of an RNA aptamer-assisted CRISPR/Cas9 system for efficiently generating and isolating Cas9-free mutants in plant”* (Manuscript ID: PGENETICS-D-25-00713) to *PLOS Genetics*. We have revised the manuscript in accordance with the comments and marked all the changes on our revised manuscript by red font. Here are the point-by-point responses for the reviewers’ comments, which are presented in blue text.

Sincerely,

Yun Zhao

**Point-by-point response to the reviewers**

**Reviewer #1 (Remarks to the Author):**

Manuscript "Development of an RNA aptamer-assisted CRISPR/Cas9 system for efficiently generating and isolating Cas9-free mutants in plant" by Liu et al. developed an RNA aptamer (3WJ-4×Bro)-based CRISPR/Cas9 system (3WJ-4×Bro/Cas9) to address the complexity and inefficiency of screening Cas9-free edited plants in plant genome editing. The study characterized the physicochemical properties of RNA aptamers in vitro, constructed the system, and validated its transformation efficiency, editing efficiency, and screening performance for Cas9-free mutants in Arabidopsis thaliana. The results showed that this system outperforms the conventional GFP/Cas9 system. The innovative application of RNA aptamers as transcriptional reporters in plant genome editing provides a new strategy for efficiently generating transgene-free edited plants, with significant theoretical and practical value. However, some result analyses require refinement. The following are some comments for discussion and consideration.

Question 1: The authors attribute the fastest fluorescence decay of 3WJ-12×Bro and the slowest decay of 3WJ-8×Bro to 'scaffold length and structural density. Is this interpretation speculative? If scaffold length were the sole determinant, why does 3WJ-4×Bro (shortest scaffold) not exhibit the slowest decay rate as one might expect? This apparent contradiction requires clarification. Are there any published literature reports were used in discussion？

We thank the reviewer for this insightful comment. We have corrected this imprecise interpretation and supplemented relevant references in discussion in our revised manuscript (see lines 284-287)

Question 2: The minimum Tm value of the aptamer is as high as 58 degrees, which indicates that the aptamers theoretically have well stability in the physiological environment of 37 degrees. The conclusion that the aptamers 'theoretically have good stability' is insufficiently rigorous. The Tm value solely reflects the melting temperature and does not directly demonstrate long-term functional stability at 37℃. We recommend that excessive subjective interpretations be avoided in the Results section; such discussions may be more appropriately addressed in the Discussion."

We agree that the statement regarding “theoretical stability at 37 °C” is speculative. In the revised manuscript, we have modified the Results section to report only the measured T_m_ value of 58 °C. This indicates that the aptamer’s folded structure is thermally stable at physiological temperature, without inferring long-term functional stability (Lines 98-99).

Question 3: Was a positive control included in the RNase A resistance assay (Fig 1h)? Please explicitly state the degradation conditions (enzyme concentration/duration).

We appreciate the reviewer’s comment. In this study, we did not include an additional positive control (e.g., complete RNA degradation by RNase A), because our primary aim was to compare the relative RNase A resistance among the three aptamers. This relative comparison allowed us to evaluate the relative stability of the aptamers under identical conditions. The treatment conditions (enzyme concentration and incubation duration) are explicitly described in the Materials and Methods section (Lines 384-388).

Question 4: Figure 2a: "RAs" may be "RAAs", "FPs" may be "GFP".

We thank the reviewer for pointing out this potential ambiguity. In Figure 2a, “RAs” was intended to represent RNA aptamers in general, and “FPs” to represent fluorescent proteins in general. In this study, we specifically used 3WJ-4×Bro, 3WJ-8×Bro, and 3WJ-12×Bro as representative RAs, and GFP as a representative FP. To avoid confusion, we have revised the figure legend to read: *“Schematic view of differences between RAs/Cas9 systems, in which RNA aptamers (3WJ-4×Bro, 3WJ-8×Bro, and 3WJ-12×Bro) are used as reporters, and the FPs/Cas9 system, in which GFP represents fluorescent proteins.”*

Question 5: Cas9 Expression Discrepancy (Fig 2c-d)，While RT-qPCR shows equivalent mRNA levels, Western blot indicates reduced Cas9 protein for GFP fusions. The explanation ("incomplete 2A cleavage") lacks direct evidence (e.g., unprocessed fusion protein not shown).

We thank the reviewer for this critical comment regarding the discrepancy between Cas9 mRNA and protein levels in the P2A-GFP constructs. In fact, in our Western blot analysis of the Cas9-P2A-GFP group, we detected not only free Cas9 protein but also the unprocessed Cas9-P2A-GFP fusion protein. Our original intention was to assess incomplete P2A cleavage by comparing the relative levels of free Cas9 across constructs. To address the reviewer’s concern, we have now included the Western blot data showing the presence of the Cas9-P2A-GFP fusion protein in the revised manuscript (Fig. 2d).

Question 6: The claim that “the 3WJ-4×Bro/Cas9 system resulted in a 25-73% increase in mutation rates at single target sites and at least a 2.5-fold increase in homozygous mutation rates in the T1 generation compared to GFP/Cas9 (Fig 5d) lacks specific numerical support. We recommend presenting comparative data directly rather than using fold-change.

We have revised the manuscript by replacing fold-change descriptions with the actual mutation and homozygous mutation rates for both target sites (Fig. 5d), providing direct numerical support without altering the study’s conclusions (Lines 238-241in the revised manuscript).

Question 7: Figure 5e is inconsistent with the results presented in Table 2. We recommend revising the wording to reduce speculative phrasing in the Results section. Additionally, certain conclusions contain instances of overinterpretation; descriptions should be modified to clearly delineate the scope of applicability for these claims.

We thank the reviewer for pointing this out. Upon checking, we found that the inconsistency between Figure 5e and Table 2 was due to an error in data processing when preparing the figure. The figure has now been corrected to accurately reflect the data in Table 2. Additionally, we have revised the Results section to remove speculative phrasing and have modified the relevant conclusions to clearly delineate the scope of applicability, avoiding overinterpretation.

**Reviewer #2 (Remarks to the Author):**

This manuscript presents an RNA aptamer-based CRISPR/Cas9 platform called 3WJ-4×Bro/Cas9 to facilitate efficient selection of Cas9-free, genome-edited plants. The authors’ system utilizes an RNA aptamer fused to the Cas9 transcript to report gene expression via fluorescence, avoiding interference with protein translation. While the manuscript introduces a potentially valuable tool, it should address the following:

1.Line 138: “Strikingly, immunoblotting revealed GFP fusion caused substantial attenuation of free Cas9 accumulation (Fig 2d).” The authors should tone down this statement, because there seems to be not so much difference in signal strength between the 3WJ-8xBro and the GFP control.

We thank the reviewer for the comment. We have revised the text in Lines 137-139 to adopt a more objective tone. Quantification of relative Cas9 expression levels normalized to β-actin (Fig. 2d) shows that the P2A-GFP fusion has lower relative expression compared with 3WJ-8×Bro, while avoiding overstated language.

2. Figure 3c and 3d: Are there any statistical indicators over the bar graphs? Related to this—line 164-167: 3WJ-4×Bro/Cas9 construct showing 5.6% transformation efficiency and GFP/Cas9 showing 5% do not seem to me as statistically significant difference.

We thank the reviewer for this important comment regarding statistical presentation and data interpretation. In response, we have added error bars representing the standard deviation of three biological replicates to Figures 3c and 3d. We also acknowledge an error in our original manuscript where a single replicate value (5.6%) was incorrectly cited for the 3WJ-4×Bro/Cas9 construct. The correct mean transformation efficiency, derived from three biological replicates, is 6.3%. This value shows a statistically significant difference (p = 0.0102) compared to the mean efficiency of the GFP/Cas9 control (5.0%). The text in Lines 167-168 has been updated to reflect the accurate mean values and the statistical significance.

3.Figure S2 is missing?

We apologize for the omission of Figure S2. It has now been added in the revised manuscript, and all supplementary materials have been carefully checked for completeness.

4.Regarding the assessment of genome editing efficiency by Sanger sequencing presented in the manuscript: While Sanger sequencing can reveal useful information about genome editing efficiency, particularly in homozygous, mono- or bi-allelic mutants, it does not provide an in-depth quantification (especially in heterozygous lines). To this end, have the authors considered using NGS for analysis of genome editing efficiency? This would provide more quantifiable data.

We appreciate the reviewer’s valuable comment. We agree that Sanger sequencing has limited quantitative ability for heterozygous mutations and may not fully capture all editing events. In this study, our primary goal was to assess the types of gene mutations, for which Sanger sequencing is reliable and sufficient^1-2^. We have considered using NGS for more precise quantification and acknowledge that it could provide deeper insights into heterozygous mutations. Due to limitations in resources and the scope of the current study, NGS was not performed; however, we plan to consider it in future work.

**References:**

1. Wang J, Chen H. A novel CRISPR/Cas9 system for efficiently generating Cas9-free multiplex mutants in *Arabidopsis*. aBIOTECH. 2019 Nov 20;1(1):6-14.

2. Xu J, Yin Y, Jian L, et al. Seeing is believing: a visualization toolbox to enhance selection efficiency in maize genome editing. Plant Biotechnol J. 2021 May;19(5):872-874.

Reviewer #3 (Remarks to the Author):

In this manuscript, Liu et al. detail the development and evaluation of a new CRISPR/Cas9 system for plant gene editing. The authors introduce the 3WJ-4×Bro/Cas9 system, which utilizes an RNA aptamer as a transcriptional reporter to address challenges in screening for Cas9-free edited plants. The study directly compares this new system to the conventional GFP/Cas9 method, demonstrating its superiority in transformation efficiency, mutation rate, and the accurate identification of transgene-free mutants. The manuscript follows up on previous work done by the lab in developing the 3WJ-4bro aptamer. While the data is promising and has potential for application in plant biology and beyond, addressing the below enumerated concerns would bolster the narrative of this manuscript.

1. Figure 1: The diagram depicting aptamer fusion (1a) is not self-explanatory and the legend does not describe the diagram. The narrative describing Fig 1e and the figure legend for it are different. Fig 1e to me does not look like a graph that shows thermal stability or degradation of aptamers at different temperatures, rather a snapshot at one condition. It would help if the authors could clarify what the figure shows and how it relates to thermal stability of the aptamers. In addition, it would be helpful to have the individual datapoints represented in all bar graphs.

We thank the reviewer for these constructive comments. In the revised manuscript, we have carefully addressed each of the raised points. The legend for Fig. 1a has been expanded to provide a more detailed and self-explanatory description of the aptamer fusion schematic. Regarding Fig. 1e, we clarify that the melting temperature (Tm) shown corresponds to the temperature at which the fluorescence of the RNA aptamer decreases by half, serving as a quantitative measure of its thermal stability. This clarification has been explicitly added to the figure legend. Furthermore, individual data points have been incorporated into all bar graphs to improve data transparency and representation.

2. Figure 2: The authors have tried to characterize performance of the RAA/Cas9 system in *N. bethamiana* but this is a transient transformation and would be mosaic. Extracting total RNA and total protein from mosaic expressing plants could confound the amount of cas9 and may account for the differences in cas9 shown by immunoblotting. Perhaps this requires some clarification in the methods and results section of this manuscript. The color scheme used for the bar chart here is different to Fig 1. Maintaining a uniform color scheme for each condition across all figures will help the reader. Figure legend says they used an n=3 for expression studies. Are these 3 different plants? Or 3 leaves from the same plant? Did each experiment have technical replicates?

We sincerely thank the reviewer for raising this critical point regarding the potential confounding effect of mosaic expression in our transient *N. benthamiana* system. To minimize the impact of mosaicism on Cas9 quantification, we implemented the following measures: (1) Targeted leaf disc sampling – leaf discs were harvested exclusively from areas exhibiting strong GFP fluorescence, thereby enriching for successfully transformed cells; (2) Biological and technical replicates – at least three independent biological replicates were analyzed, with each replicate consisting of pooled leaf discs from three independent plants; for detection, each sample was measured in three technical replicates. These clarifications have been added to the Methods section (lines 429-433, 442-443). In addition, we have updated the bar charts to maintain a uniform color scheme across all figures for consistency.

3.Figure 3: The experimental design for screening transformants is rigorous and the use of multiple assays allows for the identification of false positive and false negative screens. Statistical analysis of this screen is depicted in Fig 3b however, this figure graph is not described comprehensively in the figure legend and statistical significance is not denoted. I would like to request that the authors maintain consistency in their labeling and color scheme which would help bring their point across easily. Figures 3c, d, e, and f should have the order of conditions and colors per condition consistent among them. The authors report that growth of Arabidopsis plants is not affected by the RAA or GFP based cas9 systems, but they have not reported the parameters measured, any quantifications or even the number of plants they used to draw this conclusion. Perhaps this could be clarified in the figure legend and by including it in the methods.

Thanks for your constructive suggestions, we have expanded the legend of Fig. 3b and presented relevant statistical analyses in Figs. 3c and 3d. The order of conditions and color scheme in Figs. 3c-f have been made consistent. *Arabidopsis* growth measurements (cotyledon size and number) supporting the conclusion that RAA- and GFP-based Cas9 systems do not affect growth are now included in the revised supplementary materials (Fig. S2).

4.Figure 4: The chromatogram for homozygous mutation shows large deletion with most of the base pairs proximal to the PAM sequence. Two of the other genotyped examples shown here illustrates insertions or deletions before the PAM sequence. While this is possible, it is thought to be less frequent. Did the authors see this phenomenon in most of their mutants? if so, what does it mean for the application of this technology in plant genome engineering?

We thank the reviewer for this insightful comment regarding the distribution of edited sites. In our study, the large majority of mutations (approximately 98%, based on our sequencing data) were indeed located proximal to the PAM sequence, which is consistent with the well-established Cas9 cleavage profile and previous reports. While the examples shown in Figure 4 include both common PAM-proximal deletions and less frequent insertions or deletions upstream of the PAM, the latter were observed in only a small minority (<2%) of edited alleles. This mutation profile confirms that our 3WJ-4×Bro/Cas9 system maintains the canonical Cas9 activity, producing predictable edits primarily around the cut site 3-4 bp upstream of the PAM. The occasional upstream indels are likely the result of alternative DNA repair pathways and do not detract from the overall utility and precision of the system for most plant genome engineering applications, such as gene knock-outs or targeted trait stacking.

5.Figure 5: Authors have attempted to investigate the efficiency of isolating cas9-free mutants in the T2 generation. However, the rationale for using dual gRNAs is not clear and the conclusions drawn from this figure does not shed light on whether dual gRNAs are necessary for RAA/Cas9 system. When you say editing of Att4 results in trichome defects at T1- is this 100% and shown in all T1 plants. Furthermore, they use Fig 5c to make two different points. 1) While talking about T1 they mention Fig 5c 3WJ-4Bro/Cas9 induces homozygous mutations at both target sites, but GFP/Cas9 only in 1. 2) They point to Fig 5c to illustrate their point that only 1 homozygous mutant for both sites was found in cas9 Free T2 population. I am confused about which point 5c illustrates. If only one Homozygous mutant for both sites was found – then point 1 is not well supported. Authors should represent point 1 with other chromatogram examples. Fig 5c can only represent T1 or T2 – not both.

We appreciate the reviewer’s detailed comment. We provide the following clarifications:

1. In this study, dual gRNAs were used to evaluate the performance of 3WJ-4×Bro/Cas9 in dual-target gene editing. By targeting two sites simultaneously, we aimed to systematically assess the efficiency of this system in multi-site editing.

2. Editing of the *AtTTG1* gene resulted in a trichomeless phenotype, which was observed in 100% of the homozygous T1 mutant plants, consistent with previous reports ^[1]^. This phenotype served as a clear visual marker for successful editing.

3. We apologize for the confusion caused by the initial misrepresentation of Figure 5c. The figure is intended solely to illustrate the genotyping results of a representative T1 plant edited using the 3WJ-4×Bro/Cas9 system, demonstrating successful homozygous mutation at both target sites. It does not represent T2 data.

The data regarding the efficiency of obtaining Cas9-free, double homozygous mutants in the T2 generation are now exclusively and clearly presented in Table 2. According to this table, only one plant was identified as a double homozygous mutant without the Cas9 transgene, which is a key finding for assessing the system's efficiency in generating transgene-free, multiplex-edited plants.

**References:**

1. Zhang B, Schrader A. TRANSPARENT TESTA GLABRA 1-Dependent Regulation of Flavonoid Biosynthesis. Plants (Basel). 2017 Dec 20;6(4):65.

6. Discussion: Is only a reiteration of the results and lacks in-depth discussion about the biochemistry of these aptamers and applications in the field. This section requires some work.

We thank the reviewer for this suggestion. We have revised the discussion to include the aptamers’ biochemical properties and their potential applications (lines 321-329).

7.Statistical Analysis: You have opted to use a Student’s T test even when you are comparing more than two independent variables. Why did you opt for this instead of a One Way ANOVA. Please expand on how you did the statistical analysis in the methods section. Some legends don’t have sample sizes.

We appreciate the reviewer’s comment. We have updated the statistical analysis: all multi-group comparisons are now analyzed using one-way ANOVA followed by Tukey’s post hoc test. Figure legends have been revised accordingly, and sample sizes (n) are now included. These changes did not affect the overall conclusions of the study.

8.Figures: Please make the x axis label order of samples consistent across all figures and maintain a consistent color scheme for the samples. Show us the individual datapoints for the bar graphs.

We thank the reviewer for these suggestions. We have revised all figures to ensure that the order of samples on the x-axis is consistent across figures and that the color scheme for each sample is uniform. In addition, individual data points have been added to all bar graphs to better represent data distribution.
